# Tuberculosis — United States, 2017

**DOI:** 10.15585/mmwr.mm6711a2

**Published:** 2018-03-23

**Authors:** Rebekah J. Stewart, Clarisse A. Tsang, Robert H. Pratt, Sandy F. Price, Adam J. Langer

**Affiliations:** 1 Division of Tuberculosis Elimination, National Center for HIV/AIDS, Viral Hepatitis, STD, and TB Prevention, CDC.

In 2017, a total of 9,093 new cases of tuberculosis (TB) were provisionally* reported in the United States, representing an incidence rate of 2.8 cases per 100,000 population. The case count decreased by 1.8% from 2016 to 2017, and the rate declined by 2.5% over the same period. These decreases are consistent with the slight decline in TB seen over the past several years (*1*). This report summarizes provisional TB surveillance data reported to CDC’s National Tuberculosis Surveillance System for 2017 and in the last decade. The rate of TB among non–U.S.-born persons in 2017 was 15 times the rate among U.S.-born persons. Among non–U.S.-born persons, the highest TB rate among all racial/ethnic groups was among Asians (27.0 per 100,000 persons), followed by non-Hispanic blacks (blacks; 22.0). Among U.S.-born persons, most TB cases were reported among blacks (37.1%), followed by non-Hispanic whites (whites; 29.5%). Previous studies have shown that the majority of TB cases in the United States are attributed to reactivation of latent TB infection (LTBI) (*2*). Ongoing efforts to prevent TB transmission and disease in the United States remain important to continued progress toward TB elimination. Testing and treatment of populations most at risk for TB disease and LTBI, including persons born in countries with high TB prevalence and persons in high-risk congregate settings (*3*), are major components of this effort.

Health departments in the 50 states and the District of Columbia electronically report to CDC verified TB cases that meet the CDC and Council of State and Territorial Epidemiologists’ surveillance case definition.^†^ Reported data include the patient’s country of birth, self-identified race and ethnicity (i.e., Hispanic or non-Hispanic), human immunodeficiency virus (HIV) status, drug-susceptibility test results, and information on risk factors, including homelessness^§^ and residence in a congregate setting (i.e., long-term care or correctional facility). Persons of Hispanic ethnicity might be of any race; non-Hispanic persons are categorized as Asian, black, white, American Indian/Alaska Native, Native Hawaiian or other Pacific Islander, or of multiple races. A U.S.-born person is defined as a person who was eligible for U.S. citizenship at birth, regardless of the actual place of birth. CDC calculates overall national and state TB rates using U.S. Census Bureau population estimates and by racial/ethnic group and national origin using population denominators from the bureau’s Current Population Survey.^¶^ Yearly case counts and rates were compared overall and by origin of birth and race/ethnicity. Annual percent changes between years were calculated to compare differences in case counts and rates over time. Drug-susceptibility testing results were reported from culture-confirmed cases in 2016, the most recent year for which complete TB drug-susceptibility data were available.

State-specific TB rates (cases per 100,000 persons) ranged from 0.3 in Montana to 8.1 in Hawaii (Table 1) with a median state TB rate of 1.8. As has been the case for the past decade, four states (California, Florida, New York, and Texas) reported half of the total TB cases in the United States in 2017. The annual percent change in rate in recent years has slowed from an average decline of 5.3% during 2010–2013 to an average decline of 2.0% during 2014–2017. In 2017, a total of 6,346 (69.8%) of U.S. TB cases occurred among non–U.S.-born persons, 2,698 (29.7%) cases occurred among U.S.-born persons, and 49 (0.5%) occurred among persons with no reported national origin. The TB rate among non–U.S.-born persons (14.6) was 15 times the rate among U.S.-born persons (1.0) (Figure). Although these rates represent decreases among both groups in 2017 compared with 2016, the rate among U.S.-born persons declined 7.0%, whereas that among non–U.S.-born persons declined 0.9%.

**TABLE 1:** Tuberculosis (TB) case counts and incidence with annual percent changes, by U.S. Census division and state/district — 50 states and the District of Columbia, 2016 and 2017

Census division/State	No. of reported TB cases*			TB incidence^†^ per 100,000 persons		
	2016	2017	% change	2016	2017	% change^§^
**Division 1: New England**						
Connecticut	52	63	21.2	1.4	1.8	21.1
Maine	23	14	-39.1	1.7	1.0	-39.4
Massachusetts	190	210	10.5	2.8	3.1	9.9
New Hampshire	15	19	26.7	1.1	1.4	25.9
Rhode Island	12	13	8.3	1.1	1.2	8.1
Vermont	6	3	-50.0	1.0	0.5	-50.0
Total	298	322	8.1	2.0	2.2	7.7
**Division 2: Middle Atlantic**						
New Jersey	294	278	-5.4	3.3	3.1	-5.7
New York	758	806	6.3	3.8	4.1	6.3
Pennsylvania	173	192	11.0	1.4	1.5	10.8
Total	1,225	1,276	4.2	2.9	3.1	4.0
**Division 3: East North Central**						
Illinois	341	337	-1.2	2.7	2.6	-0.9
Indiana	109	100	-8.3	1.6	1.5	-8.7
Michigan	133	132	-0.8	1.3	1.3	-1.0
Ohio	140	150	7.1	1.2	1.3	6.8
Wisconsin	40	50	25.0	0.7	0.9	24.5
Total	763	769	0.8	1.6	1.6	0.6
**Division 4: West North Central**						
Iowa	48	47	-2.1	1.5	1.5	-2.5
Kansas	39	29	-25.6	1.3	1.0	-25.8
Minnesota	168	178	6.0	3.0	3.2	5.0
Missouri	99	87	-12.1	1.6	1.4	-12.4
Nebraska	28	20	-28.6	1.5	1.0	-29.0
North Dakota	22	14	-36.4	2.9	1.9	-36.4
South Dakota	12	14	16.7	1.4	1.6	15.6
Total	416	389	-6.5	2.0	1.8	-7.0
**Division 5: South Atlantic**						
Delaware	16	15	-6.3	1.7	1.6	-7.2
District of Columbia	25	36	44.0	3.7	5.2	42.0
Florida	639	549	-14.1	3.1	2.6	-15.4
Georgia	303	290	-4.3	2.9	2.8	-5.4
Maryland	221	208	-5.9	3.7	3.4	-6.3
North Carolina	219	213	-2.7	2.2	2.1	-3.8
South Carolina	102	101	-1.0	2.1	2.0	-2.3
Virginia	203	204	0.5	2.4	2.4	-0.2
West Virginia	14	16	14.3	0.8	0.9	15.1
Total	1,742	1,632	-6.3	2.7	2.5	-7.3
**Division 6: East South Central**						
Alabama	112	120	7.1	2.3	2.5	6.8
Kentucky	91	65	-28.6	2.1	1.5	-28.9
Mississippi	61	53	-13.1	2.0	1.8	-13.1
Tennessee	103	128	24.3	1.5	1.9	23.0
Total	367	366	-0.3	1.9	1.9	-0.8
**Division 7: West South Central**						
Arkansas	91	85	-6.6	3.0	2.8	-7.1
Louisiana	127	141	11.0	2.7	3.0	11.1
Oklahoma	78	54	-30.8	2.0	1.4	-30.9
Texas	1,250	1,127	-9.8	4.5	4.0	-11.1
Total	1,546	1,407	-9.0	3.9	3.5	-10.0
**Division 8: Mountain**						
Arizona	188	188	0.0	2.7	2.7	-1.5
Colorado	64	84	31.3	1.2	1.5	29.4
Idaho	18	9	-50.0	1.1	0.5	-51.1
Montana	4	3	-25.0	0.4	0.3	-25.8
Nevada	55	80	45.5	1.9	2.7	42.6
New Mexico	39	37	-5.1	1.9	1.8	-5.2
Utah	20	29	45.0	0.7	0.9	42.3
Wyoming	1	2	100.0	0.2	0.3	101.9
Total	389	432	11.1	1.6	1.8	9.5
**Division 9: Pacific**						
Alaska	57	52	-8.8	7.7	7.0	-8.6
California	2,059	2,056	-0.1	5.2	5.2	-0.8
Hawaii	119	116	-2.5	8.3	8.1	-2.4
Oregon	70	69	-1.4	1.7	1.7	-2.8
Washington	205	207	1.0	2.8	2.8	-0.7
Total	2,510	2,500	-0.4	4.8	4.7	-1.2
**United States**	**9,256**	**9,093**	**-1.8**	**2.9**	**2.8**	**-2.5**

* Case counts based on data from the National Tuberculosis Surveillance System as of February 12, 2018.

^†^ U.S. Census Bureau midyear population estimates provide the denominators used to calculate TB incidence.

^§^ Percentage change in incidence is calculated based on unrounded incidence for 2016 and 2017.

**FIGURE:**
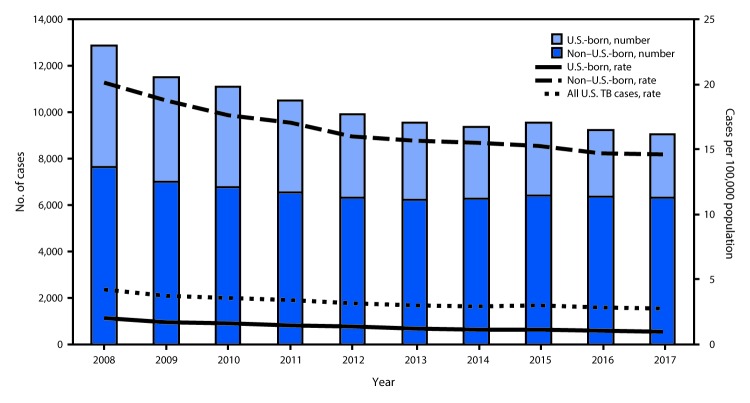
Number of tuberculosis (TB) cases and rate, by national origin — United States, 2008–2017

Among non–U.S.-born persons, the highest TB rate among all racial/ethnic groups occurred among Asians (27.0 per 100,000 persons), followed by blacks (22.0) (Table 2). As in previous years, in 2017, the top five countries of birth of non–U.S.-born persons with TB were Mexico (1,204; 19.0% of all non–U.S.-born persons with TB), Philippines (783; 12.3%), India (595; 9.4%), Vietnam (526; 8.3%), and China (400; 6.3%). Persons who received a diagnosis of TB ≥10 years after arriving in the United States accounted for 2,854 (45.0%) of all TB cases among non–U.S.-born persons.

**TABLE 2:** Tuberculosis (TB) case counts and incidence,* by national origin and race/ethnicity — United States, 2014–2017^†^

U.S. population group	No. of cases (incidence)			
	2014	2015	2016	2017
**U.S.-born^§^**				
Hispanic	651 (1.8)	659 (1.8)	602 (1.6)	589 (1.5)
White, non-Hispanic	969 (0.5)	985 (0.5)	910 (0.5)	797 (0.4)
Black, non-Hispanic	1,185 (3.4)	1,141 (3.3)	1,067 (3.0)	1,001 (2.8)
Asian	139 (2.1)	139 (2.1)	146 (2.1)	136 (2.0)
American Indian/Alaska Native	117 (5.2)	144 (7.0)	108 (5.0)	89 (3.7)
Native Hawaiian/Pacific Islander	40 (6.0)	42 (6.1)	31 (4.3)	45 (6.5)
Multiple or unknown race/ethnicity	28 (*—*^¶^)	25 (—^¶^)	25 (—^¶^)	41 (—^¶^)
Total U.S.-born	3,129 (1.1)	3,135 (1.1)	2,889 (1.0)	2,698 (1.0)
**Non–U.S.-born**				
Hispanic	2,095 (11.2)	2,036 (10.4)	1,988 (10.0)	1,952 (9.9)
White, non-Hispanic	276 (3.6)	257 (3.4)	286 (3.8)	268 (3.5)
Black, non-Hispanic	829 (23.6)	855 (23.1)	908 (22.6)	892 (22.0)
Asian	2,945 (29.6)	3,156 (29.6)	3,055 (27.2)	3,087 (27.0)
American Indian/Alaska Native	0 (0.0)	1 (1.9)	1 (2.9)	3 (4.3)
Native Hawaiian/Pacific Islander	51 (22.8)	60 (18.6)	47 (13.0)	62 (21.0)
Multiple or unknown race/ethnicity	69 (—^¶^)	42 (—^¶^)	70 (—^¶^)	82 (—^¶^)
Total non–U.S.-born	6,265 (15.5)	6,407 (15.3)	6,355 (14.7)	6,346 (14.6)
**Unknown national origin**	5 (—^¶^)	6(—^¶^)	12 (—^¶^)	49 (—^¶^)
**Overall total**	**9,399 (2.9)**	**9,548 (3.0)**	**9,256 (2.9)**	**9,093 (2.8)**

* Incidence calculated per 100,000 persons.

^†^ Case counts based on data from the National Tuberculosis Surveillance System as of February 12, 2018. The Current Population Survey (https://www.census.gov/programs-surveys/cps.html) provides the population denominators used to calculate TB incidence according to national origin and racial/ethnic group.

^§^ U.S.-born persons were born in the United States or U.S. territories (American Samoa, Commonwealth of the Northern Mariana Islands, Guam, Puerto Rico, and the U.S. Virgin Islands) or born elsewhere to a U.S. citizen. Non–U.S.-born persons were born outside the United States (or the U.S. territories), and include those born in the sovereign freely associated states (Federated States of Micronesia, Republic of the Marshall Islands, and Republic of Palau) (unless one or both parents were U.S. citizens).

^¶^ Incidence was not calculated for these categories.

Among U.S.-born persons in 2017, a total of 1,001 (37.1%) TB cases were reported among blacks, and 797 (29.5%) among whites, representing a 55% decrease in case count for each group in the past decade. The highest TB rate among U.S.-born persons was reported among Native Hawaiians and other Pacific Islanders (6.5), followed by American Indians and Alaska Natives (3.7), blacks (2.8), Asians (2.0), Hispanics (1.5), and whites (0.4).

In 2017, 388 (4.3%) TB cases were reported among persons experiencing homelessness in the year preceding diagnosis, 148 (1.6%) among persons residing in a long-term care facility at the time of diagnosis, and 266 (3.0%) among persons confined in a correctional facility at the time of diagnosis. Although cases among U.S.-born persons accounted for <30% of total TB cases in the United States, they accounted for 61.1% among those reporting homelessness, 44.6% among those in long-term care facilities, and 39.5% among persons incarcerated at the time of diagnosis. HIV status was known for 86.3% of TB cases reported in 2017; among those cases, 5.6% had coinfection with HIV.

Drug susceptibility testing results were reported for 98.3% of culture-confirmed cases in 2016. Among all 9,256 cases reported in 2016, 97 (1.0%) were multidrug-resistant (MDR) TB, including 78 (80.4%) cases with primary MDR TB,** 18 (18.6%) with a prior history of TB, and one (1.0%) with an unknown history of previous TB diagnosis. Among the 97 MDR TB cases in 2016, 89 (91.8%) occurred among 6,355 non–U.S.-born persons, accounting for 1.4% of all TB cases among non–U.S.-born persons. One case of extensively drug-resistant^††^ TB was reported in a non–U.S.-born person.

## Discussion

In 2017, the provisional TB case count and incidence were the lowest in the United States since national TB surveillance began in 1953 (*1*); however, the rate in 2017 (2.8 per 100,000) is still 28 times the U.S. elimination threshold of less than one case per million persons (*4*). Since 2014, the annual percentage change in rate compared with the preceding year has slowed to an average decline of 2.0%. To achieve TB elimination by 2100, a sustained annual decline of 3.9% is required.^§§^ Previous studies have indicated that reactivation of LTBI, rather than recent transmission, is the primary driver of TB disease in the United States, accounting for >80% of all TB cases (*2*). Ongoing efforts to prevent TB transmission must be sustained, and efforts to detect and treat LTBI, especially among groups at high risk, must be increased.

An epidemiologic model found that substantial (i.e., quadruple) increases in LTBI testing and treatment completion would accelerate progress toward TB elimination (*4*). Several accepted treatment regimens are available for LTBI (*5*). Among these, CDC encourages the use of shorter, rifamycin-based regimens, such as 4 months of rifampin or 3 months of once-weekly rifapentine plus isoniazid, which have better treatment completion rates (*6*) and are less hepatotoxic (*7*,*8*) than a regimen of 9 months of isoniazid. Improved treatment completion, less toxicity, and shorter treatment regimens can reduce morbidity and accelerate TB elimination in the United States.

Distinct disparities exist between populations affected by TB. Highly affected and vulnerable populations include persons housed in congregate settings and persons from countries with high TB prevalences. The U.S. Preventive Services Task Force (USPSTF) recommends screening for LTBI in populations at increased risk, including persons born in countries with high TB prevalences, regardless of length of residence in the United States and age (*3*); this recommendation is consistent with a previously published report documenting an increasing proportion of TB diagnoses among non–U.S.-born persons living in the United States for ≥10 years (*9*). In addition to USPSTF screening recommendations, CDC also recommends treatment of LTBI to reduce the number of persons developing TB disease (*5*). Increased support of global TB elimination efforts would help to reduce global TB and LTBI prevalence, thereby indirectly reducing the incidence of reactivation TB in the United States among non–U.S.-born persons from higher-prevalence countries.

Spending time in congregate settings, such as homeless shelters, long-term care facilities, and correctional facilities, increases the risk for TB transmission. Most requests from state or local health departments for on-site CDC assistance arise from TB outbreaks involving congregate settings serving vulnerable populations (*10*). The USPSTF recommends TB testing for persons who have lived in high-risk congregate settings, such as homeless shelters and correctional facilities (*3*). Control of transmission requires not only preventing disease through treatment of LTBI, but also strong infection control practices in settings with increased risk for transmission.

The findings in this report are subject to at least two limitations. First, this analysis is limited to reported provisional TB cases and case rates for 2017; final results will be available in the fall of 2018. Second, case rates are calculated using 2017 population estimates as denominators.

Since 2015, TB case counts and rates in the United States have declined, in large part because of the work of local TB programs in detecting and treating persons with TB disease. Approximately 96% of persons with diagnosed TB disease in the United States complete therapy (*1*), thereby limiting the risk for further transmission and development of MDR TB. TB is preventable through LTBI testing and treatment and implementation of effective infection control measures; however, TB elimination goals in the United States will not be achieved without steadfast engagement among public health partners and sustained prevention and control programs. Public health priorities for TB elimination in the United States include developing comprehensive and innovative approaches to diagnosing, treating, and monitoring LTBI; continued engagement by the United States in global TB control efforts; and enhanced efforts to prevent TB transmission in the United States, particularly in congregate settings.

**Summary**

**What is already known about this topic?**

Since 1993, tuberculosis (TB) case counts and rates have declined in the United States. As the number of cases decreases overall, an increasing percentage of cases occurs among non–U.S.-born persons. Disparities also exist within racial, ethnic, and social groups among U.S.-born persons with TB.

**What is added by this report?**

In 2017, preliminary data indicate that 9,093 new TB cases were reported in the United States, a rate of 2.8 per 100,000 population. This is the lowest case count and rate on record, representing a decrease in case count of 1.8% from 2016 to 2017 and a 2.5% decrease in rate over the same period. The annual percent decline in rate in recent years has slowed to 2.0%. To achieve TB elimination by 2100, a sustained annual decline of 3.9% is required.

**What are the implications for public health practice?**

Control of active TB and a major effort to decrease latent TB infection are both necessary to reduce morbidity and achieve TB elimination in the United States. An important component of this strategy is the testing and treatment of populations most at risk for latent TB infection, persons born in countries with high TB prevalence, and persons in high-risk congregate settings.

## Footnotes

* This report is limited to National Tuberculosis Surveillance System case reports verified as of February 12, 2018. Updated data will be available in CDC’s annual TB surveillance report later this year.

† Appendix A, page 137, Report of Verified Case of Tuberculosis (RVCT) Instruction Manual. https://www.cdc.gov/tb/programs/rvct/instructionmanual.pdf.

§ Homelessness is defined as a lack of fixed, regular, and adequate nighttime residence at any time during the 12 months preceding TB diagnostic evaluation and a primary nighttime residence that is either a shelter, an institution that provides temporary residence for persons intended to be institutionalized, or a public place not designated for, or ordinarily used as, a regular sleeping accommodation for human beings. A homeless person may also be defined as a person without a home or in an unstable housing situation. Pages 85–86, Report of Verified Case of Tuberculosis (RVCT) Instruction Manual. https://www.cdc.gov/tb/programs/rvct/instructionmanual.pdf.

¶ U.S. Census Bureau Population and Housing Unit Estimates Tables; https://www.census.gov/programs-surveys/popest/data/tables.html and Current Population Survey; https://www.census.gov/programs-surveys/cps.html.

** Primary multidrug-resistant tuberculosis (TB) is defined as a case of TB in a person with a *Mycobacterium tuberculosis* isolate with resistance to at least isoniazid and rifampin and who was not previously diagnosed with or treated for drug-susceptible TB disease.

†† Defined by the World Health Organization as a case of TB in a person with a *Mycobacterium tuberculosis* isolate with resistance to at least isoniazid and rifampin among first-line anti-TB drugs, resistance to any fluoroquinolone (e.g., ciprofloxacin or ofloxacin), and resistance to at least one second-line injectable drug (i.e., amikacin, capreomycin, or kanamycin).

§§ Sustained annual percent decline to reach TB elimination calculated as the yearly incidence reduction necessary to get from current rate to one case per million persons in 2100.

## References

[1] CDC. Reported tuberculosis in the United States, 2016. Atlanta, GA: US Department of Health and Human Services, CDC; 2017.

[2] Yuen CM, Kammerer JS, Marks K, Navin TR, France AM. Recent transmission of tuberculosis—United States, 2011–2014. PLoS One 2016;11:e0153728. . 10.1371/journal.pone.0153728 27082644 PMC4833321

[3] Bibbins-Domingo K, Grossman DC, Curry SJ, ; US Preventive Services Task Force. Screening for latent tuberculosis infection in adults: US Preventive Services Task Force recommendation statement. JAMA 2016;316:962–9. 10.1001/jama.2016.11046 27599331

[4] Hill AN, Becerra J, Castro KG. Modelling tuberculosis trends in the USA. Epidemiol Infect 2012;140:1862–72. 10.1017/S095026881100286X 22233605

[5] CDC. Tuberculosis (TB) treatment. Atlanta, GA: US Department of Health and Human Services, CDC; 2016. https://www.cdc.gov/tb/topic/treatment/default.htm

[6] McClintock AH, Eastment M, McKinney CM, Treatment completion for latent tuberculosis infection: a retrospective cohort study comparing 9 months of isoniazid, 4 months of rifampin and 3 months of isoniazid and rifapentine. BMC Infect Dis 2017;17:146. 10.1186/s12879-017-2245-8 28196479 PMC5310079

[7] Bliven-Sizemore EE, Sterling TR, Shang N, ; TB Trials Consortium. Three months of weekly rifapentine plus isoniazid is less hepatotoxic than nine months of daily isoniazid for LTBI. Int J Tuberc Lung Dis 2015;19:1039–44, i–v. 10.5588/ijtld.14.0829 26260821 PMC5080618

[8] Menzies D, Long R, Trajman A, Adverse events with 4 months of rifampin therapy or 9 months of isoniazid therapy for latent tuberculosis infection: a randomized trial. Ann Intern Med 2008;149:689–97. 10.7326/0003-4819-149-10-200811180-00003 19017587

[9] Tsang CA, Langer AJ, Navin TR, Armstrong LR. Tuberculosis among foreign-born persons diagnosed ≥10 years after arrival in the United States, 2010–2015. MMWR Morb Mortal Wkly Rep 2017;66:295–8. 10.15585/mmwr.mm6611a3 28333913 PMC5657888

[10] Mindra G, Wortham JM, Haddad MB, Powell KM. Tuberculosis outbreaks in the United States, 2009–2015. Public Health Rep 2017;132:157–63. 10.1177/0033354916688270 28147211 PMC5349481

